# Progress in Gynæcology

**Published:** 1897-05-01

**Authors:** 


### Progress in Gynæcology.

(*Concluded from page* 64.)

**Anæsthesia in Labour.**—Sobrouvaroff[13] never pushes chloroform to the stage of surgical anæsthesia in physiological labour, and would never administer it until true labour pains set in. He considers ether inferior, and that it should only be used for a short period. He finds the best calmative for primiparæ to be a hypodermic injection of hydrochlorate of morphia, gr. ⅙. He also employs hydrochlorate of cocain locally as an adjunct. He finds that bromide of ethylene, nitrous oxide, and other anæsthetics are unsatisfactory; he now adopts a mixed method, namely, a hypodermic injection of morphia followed by cocain locally and chloral by the mouth.

The following conclusions are arrived at by Bukoewsky[14]: (1) That ether lessens pain and can make labour painless. (2) That it does not prolong labour; but, in primiparæ, lessens the average duration of labour 54 minutes. (3) It increases the efficiency of uterine contractions, does not shorten the length of the pains, but shortens the pauses between the pains. (4) Does not influence unfavourably the puerperal period, the lochia becoming colourless earlier after its administration during labour. (5) Its use does not create a tendency to hæmorrhage. (6) Involution is not interfered with, nor pulse respiration or temperature. (7) Formation of milk sometimes delayed for 24 hours after its use. (8) It does not affect the fœtus unfavourably. The best time for its administration is when the os admits three fingers.

In criticising recorded cases of death from chloroform anæsthesia during labour, Ballantyne[15] said that there was no proof in any one of them that the anæsthetic was administered by a capable person; in one case, at any rate, there was gross maladministration. Idiosyncracy with regard to it might be present, and impurities were a source of danger. The practical immunity of the parturient woman is explicable by—first, the pharmacological action of the drug; secondly, the physiological condition of the woman in labour; and thirdly, the action of the drug on the modified physiological state. The important points of the pharmacological action are: (1) Increased, then suspended sensation; (2) suspended reflex action; (3) paralysis of vasomotor and respiratory centres, with lowering of blood pressure and slowing of respiration; and (4) paralysis of cardiac ganglia.

The physiological modifications in the parturient woman were: (1) General increase of valvular tension; (2) increase of total mass of blood in the system; (3) eccentric hypertrophy of heart. In active labour the chief phenomena were: (1) Intermitting muscular contraction of diaphragm, abdominal muscles, and uterus, with compression of blood vessels of abdomen; (2) accelerated pulse-rate; (3) forced expiration and costal breathing. Hence results high blood pressure and augmented respiratory activity caused by physiological "artificial respiration" induced by labour pains. The respiratory centre is no doubt stimulated by dilatation of the passages by the advancing fœtus in the same way as is done by forcible dilatation of the sphincter ani.

Chloroform prevents shock and lessens exhaustion in obstetric operations and long labours. Its use prevented dangers arising from brittle blood-vessels, and by controlling muscular action and minimising blood pressure saves lives in heart disease. The only contra-indications were pulmonary emphysema and cases of rapid and great hæmorrhage.

Conclusions: (1) That the method of administration should be as simple as possible, but care and watchfulness are all important; (2) that air should at first be freely admitted; (3) attention persistently directed to the respiration; (4) that in normal labour chloroform should only be administered during a pain, except when the head was passing the outlet; and (5) the administration should be stopped after the birth of the head.

**Adherent Placenta.**—That the only effective treatment of firmly adherent placenta is by thorough curetting of the uterus is the opinion of Nyulasy.[16] He considers that the method of waiting for the discharge of retained portions and treating the patient meanwhile with antiseptic injections is mere tinkering, and fraught with grave danger to the patient; the greatest risk is from the development of fulminating sepsis. Playfair supports this opinion, but considers that curetting should not be performed except by those tolerably expert in obstetric manipulations. Lusk, though not in favour of curetting, always employs it in cases of adherent placenta, and considers it successful in proportion to the promptitude and thoroughness of its application. The author describes two groups of cases. In the first group were four cases where the placenta was adherent; one was a four and a half months' abortion, one a case of seven months' premature labour, two were full time labours. In the full time cases the placenta and membranes were reported complete in one and doubtful in the other. In every case the curette removed portions of adherent placenta, and every case recovered. In the second group the placenta was expressed in each case. In two cases the placenta and membranes were reported "complete," and in one "doubtful." The cases reported "complete" were not immediately curetted. All three cases developed purulent endometritis. In all three the curette brought away fibrinous matter. One case was curetted twice on the fifth and ninth days; one three times on the sixth, eighth, and seventeenth days; and one three times on the eighth, tenth, and seventeenth days. All recovered. He quotes Krönig, who observes that in purulent endometritis "washing out of the uterus brings no benefit."

**Ante-Partum Hæmorrhage.**—Cases of ante-partum hæmorrhage without vicious insertion or premature detachment of the placenta being discoverable on examination after delivery are described by Budin.[17] The hæmorrhage is caused by rupture of the circular sinus of the placenta. In most of these cases clot is found adherent to the membranes and continuous with the border of the placenta, concealed hæmorrhage is more frequent than apparent. Insertion of the placenta in the lower uterine segment predisposes to this accident. Sudden movements, jolts, fatigue, or emotional disturbance are probable causes. The diagnosis is difficult until the placenta is expelled, and the prognosis is grave in all cases. The treatment is that of accidental hæmorrhage, namely, rupture of the membranes and delivery as soon as sufficient dilatation is present by forceps or version. De Ribes' bag should be used if dilatation is insufficient to allow of fairly rapid delivery.

**Rate of Growth of Cystic Tumours of Ovary and Broad Ligament.**—In a clinical lecture given at St. Mary's Hospital, Handfield Jones,[18] after mentioning the ordinary symptoms of ovarian and broad ligament cysts, quoted three interesting cases of exceptionally rapid growth of such cysts. In the first, an unmarried girl sought advice for general weakness and pain after food, with slight leucorrhœa. On examination of the abdomen, slight tenderness over the pelvic inlet was the only abnormality felt, and so no vaginal examination was made. Ten days later there was found dulness extending to a little above the umbilicus, which was due to a fluctuating tumour extending to four fingers' breadth from the middle line on each side. This was proved to be an ovarian tumour. He pointed out the difficulty that might occur if such a case were seen by two medical men, the one at the time when the tumour was intrapelvic, and the other at the end of a fortnight, when the tumour was above the umbilicus, and mentioned such a case, where the second medical man asserted that the first had overlooked the tumour.

In the second case, a solid tumour of the left ovary of the size of a cocoanut was found, while the right ovary was about the size of a walnut and irregular. Nothing took place for twelve months, when in six weeks the abdomen was distended by a tumour the size of the pregnant uterus, which was proved to be a multilocular ovarian cyst. Another case occurred after parturition, no swelling being noticed while the placenta was being expelled, although a little difficulty in defining the uterus was noticed by the medical man in attendance. The next day there was slight distension and dulness up to midway between umbilicus and pubes; the next day dulness up to the umbilicus; the next to midway between umbilicus and xiphoid; the next, the day of operation, up to the xiphoid cartilage. A large multilocular cyst was removed containing effused blood in the cyst walls and bloody serum in the secondary cysts; the tumour increased its size by a third in less than 24 hours.

In another case, after curetting, it was noted that the right ovary and tube formed a mass the size of a walnut, while the left ovary and tube formed one the size of a Tangerine orange. Fourteen days later the ovaries and tubes were removed. The tubes both contained between five and six ounces of sero-pus. The difference between the rapid increase of an ovarian cyst with twisted pedicle and the rapid refilling of a parovarian cyst after rupture and slow emptying was pointed out; and also the symptoms, the chief of which was pain with rapid deterioration of the patient's health. A certain degree of rise of temperature often favoured the opinion that peritonitis was present, but for the fact that the abdominal muscles were not so rigid as they are in peritonitis.

[13] Vratch. (quoted Epit. B. M. J., Dec. 26, 1896). [14] Mönatoschrift für Geburt, und Gynäkolog. (quoted Indian Med. Chir. Review, Sept. 1896. [15] Lancet, Nov. 28, 1896). [16] Intercolonial Med. Journ. of Australasia, March 20, 1896; Therapeutic Gazette, Sept. 15, 1896. [17] Presse Medicale, August 1896. [18] Practitioner, Dec., 1896.

